# First‐generation genome editing in potato using hairy root transformation

**DOI:** 10.1111/pbi.13376

**Published:** 2020-04-16

**Authors:** Nathaniel M. Butler, Shelley H. Jansky, Jiming Jiang

**Affiliations:** ^1^ United States Department of Agriculture‐Agricultural Research Service Vegetable Crops Research Unit Madison Wisconsin USA; ^2^ Department of Horticulture University of Wisconsin Madison Wisconsin USA; ^3^ Department of Plant Biology Department of Horticulture Michigan State University East Lansing Michigan USA

**Keywords:** plant transformation, CRISPR, Cas, reagent delivery, *Agrobacterium rhizogenes*, gene editing, crop, targeted mutagenesis, germ‐line, Trex2, Csy4

## Abstract

Genome editing and *cis*‐gene breeding have rapidly accelerated crop improvement efforts, but their impacts are limited by the number of species capable of being genetically transformed. Many dicot species, including some vital potato relatives being used to accelerate breeding and genetics efforts, remain recalcitrant to standard *Agrobacterium tumefaciens*‐based transformation. Hairy root transformation using *Agrobacterium rhizogenes* (*A. rhizogenes*) provides an accelerated approach to generating transgenic material but has been limited to analysis of hairy root clones. In this study, strains of *A. rhizogenes* were tested in the wild diploid potato relative *Solanum chacoense*, which is recalcitrant to infection by *Agrobacterium tumefaciens*. One strain of *A. rhizogenes* MSU440 emerged as being capable of delivering a T‐DNA carrying the GUS marker and generating transgenic hairy root clones capable of GUS expression and regeneration to whole plants. CRISPR/Cas9 reagents targeting the potato *PHYTOENE DESATURASE* (*StPDS*) gene were expressed in hairy root clones and regenerated. We found that 64%–98% of transgenic hairy root clones expressing CRISPR/Cas9 reagents carried targeted mutations, while only 14%–30% of mutations were chimeric. The mutations were maintained in regenerated lines as stable mutations at rates averaging at 38% and were capable of germ‐line transmission to progeny. This novel approach broadens the numbers of genotypes amenable to *Agrobacterium*‐mediated transformation while reducing chimerism in primary events and accelerating the generation of edited materials.

## Introduction

Genetic transformation has become a bottleneck for genome editing and *cis*‐gene breeding in crop species. As sequence‐specific nuclease (SSN) technology continues to improve, the development of new approaches to genetic transformation has lagged but is needed to implement genome editing in recalcitrant crop species (Yin *et al.*, 2017; Zhang *et al.*, 2018). A major breakthrough in monocot plant transformation was made with the development of the *Baby boom*/*Wuschel* transformation system, expanding the range of maize (*Zea mays*), sorghum (*Sorghum bicolor*), sugarcane (*Saccharum officinarum*) and rice (*Oryza sativa var. indica*) genotypes that can be transformed (Lowe *et al.*, 2016). Hairy root transformation using *Agrobacterium rhizogenes* provides a rapid approach to generating transgenic materials and additional strains that can be tested to overcome recalcitrance in primarily dicot species; however, no studies to date have analysed materials at the whole‐plant level (Alok *et al.*, 2018; Ron *et al.*, 2014). Recalcitrance across certain crop species and the need to generate edited material in less time warrants further development of plant transformation technologies.

Potato is largely a polyploid crop, with autotetraploid (2*n* = 4*x* = 48) genotypes dominating agricultural settings and breeding and genetics efforts (Hirsch *et al.*, 2013). Most cultivated, autotetraploid varieties of potato are of the subspecies ‘*tuberosum’* (*Solanum tuberosum ssp*. *tuberosum*) and can be readily transformed with *Agrobacterium tumefaciens*‐based plant transformation protocols (Halterman *et al.*, 2015). The amenability of autotetraploid varieties of potato to *Agrobacterium*‐mediated plant transformation enabled potato to be one of the first commercialized genetically modified (GM) crops. However, autotetraploid potato varieties are genetically highly heterozygous and are often highly sterile, thus are not ideal for genetic analysis or functional genomics studies. It is highly challenging to mutate all four alleles using SSNs and chimerism seen in primary events using SSNs in conventional *Agrobacterium tumefaciens*‐based protocols have further slowed genetic and genome editing efforts, respectively (Butler and Douches, 2016; Dangol et al., 2019).

A rediscovery of self‐compatible diploid (2*n* = 2*x* = 24) species of potato has provided a new opportunity to fix important traits and accelerate genetic and genomic efforts in potato (Birhman and Hosaka, 2000; Jansky *et al.*, 2016; Marand *et al.*, 2019). Most wild, diploid species of potato are self‐incompatible and are incapable of inbreeding. However, self‐compatible accessions of *Solanum chacoense* (*S. chacoense*) have been discovered and are capable of setting selfed seed without intervention (Jansky *et al.*, 2014). One such inbred line of *S. chacoense*, called ‘M6’ (Jansky *et al.*, 2014), has been crossed to the homozygous, doubled‐monoploid (‘DM’) potato (Potato Genome Sequencing Consortium, 2011), which was used to construct the potato reference genome. The resulting F1 hybrid (DMF1) is self‐compatible and has been used to develop the first inbred line‐derived F2 population in potato (Endelman and Jansky, 2016). Recently, the genome of M6 was also sequenced, providing additional genetic resources for lines derived from M6 and raising the importance of inbred lines for functional genomics (Leisner *et al.*, 2018). Nevertheless, plant transformation protocols have not yet been established for DMF1 and other related self‐compatible, diploid germplasm in potato.

In this study, a hairy root plant transformation protocol was used to generate stable targeted mutations in the DMF1 genotype in the first generation. Targeted mutations in regenerated lines were transmitted through the germ‐line to progeny and supported a clear phenotype. This demonstrates not only an advance in plant transformation of a recalcitrant crop species, but also an accelerated approach to genome editing.

## Results and Discussion

### *Agrobacterium*‐mediated transformation using a self‐compatible, diploid potato

**Figure 1:**
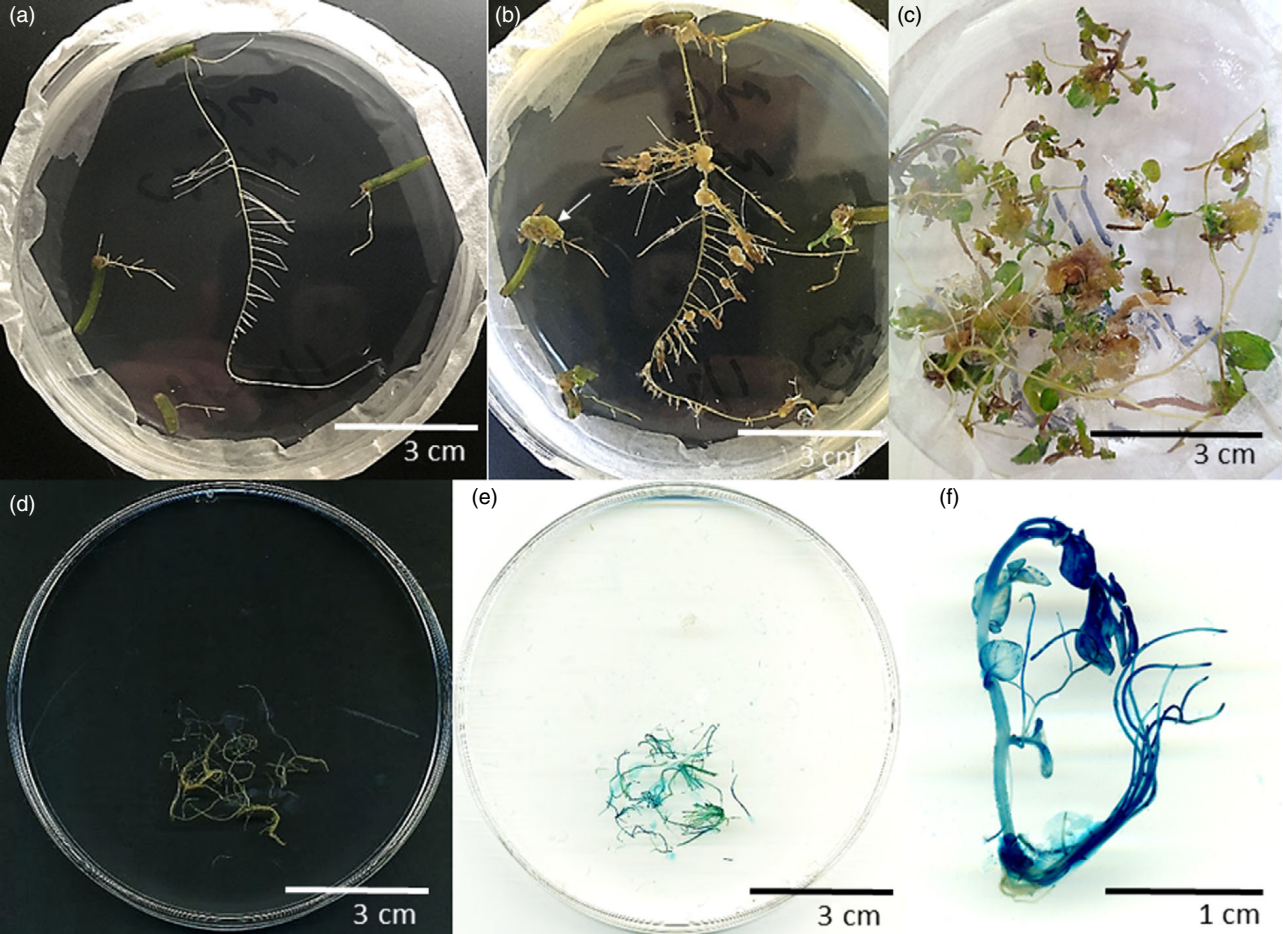
Hairy root transformation and regeneration in potato. (a) Hairy root clones derived from individual stem explants infected with *A. rhizogenes* MSU440. (b) Intermediate calli derived from hairy root clones on callus‐inducing media. White arrow indicates friable, compact callus recalcitrant to regeneration. (c) Regenerated lines originating from intermediate calli in response to cytokinin and auxin hormones in the media. (d) Transgenic hairy root clone expressing GUS reporter originating from pCAMBIA1301 (e stained). (f) GUS expression in regenerated line originating from a hairy root clone. All images are from DMF1 genotype.

*Agrobacterium*‐mediated transformation using different strains and species of *Agrobacterium* was tested to transform DMF1 (Table 1). Two strains of *A. tumefaciens* (GV3101 and LBA4404) were tested along with five strains of *A. rhizogenes* (15 834, A4, ARqua1, K599 and MSU440). Each strain carried the binary vector, pCAMBIA1301 (GenBank: AF234297.1), which constitutively expresses the β‐glucuronidase (GUS) reporter and confers hygromycin resistance in transformed explants (Jia and Wang, 2014). Successful transformation was determined by hairy root (Figure 1a) or shoot organogenesis (Figure 1c) on increasing levels of hygromycin selection, with 5 mg/L hygromycin being the threshold for wild‐type resistance (Table 1; no infection).

**Table 1:** Susceptibility of potato to different stains and species of *Agrobacterium*

	% explants with roots or shoots* (total # explants)				
	(No selection)	Hygromycin 2	Hygromycin 5	Hygromycin 8	Hygromycin 10
*A. tumefaciens* GV3101	**94%** (32)*	**20%** (40)*	**25%** (40)*	0% (33)*	0% (34)*
*A. tumefaciens* LBA4404	**92%** (35)*	**18%** (42)*	**15%** (38)*	0% (30)*	0% (36)*
*A. rhizogenes* A4	**26%** (38)	**20%** (20)	**36%** (22)	0% (20)	0% (22)
*A. rhizogenes* MSU440	**21%** (28)	**70%** (20)	**36%** (22)	**18%** (22)	**9%** (22)
*A. rhizogenes* ARqua1	**3%** (38)				
*A. rhizogenes* 15834	0% (42)				
*A. rhizogenes* K599	0% (43)				
(no infection)	**93%** (27)*	**15%** (26)*	**17%** (30)*	0% (28)*	0% (26)*

Stem explants (# in parentheses) were infected with a strain of *Agrobacterium* (far left column) and put on either MS20 media (‘*A. rhizogenes’* strains) or regeneration media (‘*A. tumefaciens’* strain) with different levels of hygromycin selection (in mg/L). Resulting explants with regenerated shoots (values with asterisks) or hairy root clones were counted and are shown as a per cent of the total stem explants. Percentages higher than zero are bolded. Data are from the DMF1 genotype.

*Agrobacterium tumefaciens* stains failed to produce explants capable of survival beyond wild‐type levels of selection (Table 1). These results reflect similar experiments using M6 (data not shown), suggesting M6 and DMF1 are recalcitrant to the tested *A. tumefaciens* strains. However, *A. rhizogenes* strain MSU440 generated explants capable of survival and the production of hairy root clones under selection up to 10 mg/L hygromycin for DMF1 (Table 1) and 8 mg/L for M6 (data not shown). Hairy root clones from DMF1 explants infected with MSU440 were further validated as being transgenic using GUS staining, with hairy root clones from A4 used as a negative control (Table S2). Hairy root clones from MSU440 explants expressed GUS activity originating from all levels of selection excluding 10 mg/L hygromycin, whereas no GUS activity was detected in A4 hairy root clones. These results suggest *A. rhizogenes* strain MSU440 is capable of transforming DMF1 and overcoming recalcitrance observed with *A. tumefaciens* stains, whereas strains such as A4 are largely ineffective for binary T‐DNA delivery.

### Regeneration of hairy root clones and expression of transgene‐mediated traits

Hairy root transformation has traditionally been used to readily generate transformed material in the form of hairy root clones, which can be easily manipulated and grow quickly (Veena and Taylor, 2007). However, the creation of hairy root clones as the material of study restricts analysis of non‐root plant traits. To overcome this, regeneration of individual hairy root clones was attempted using GUS‐expressing DMF1 hairy root clones (Figure 1b‐c). Regeneration was accomplished by transferring hairy root clones to regeneration media which supported callus formation (Figure 1b) and shoot organogenesis (Figure 1c), typical of conventional *A. tumefaciens* transformation. Interestingly, not every hairy root clone was capable of regeneration on the media used (Table 2; 14%–100%) and instead formed friable or compact callus (Figure 1b; arrow). The great variation in regeneration capabilities across hairy root clones seemed to be influenced by type of callus formed, where the callus observed on root explants capable of regeneration appeared to be less compact and friable (Figure 1c). In either case, the dominant form of callus on root explants seemed to be auxin‐ and cytokinin‐mediated since wounding had only minimal effects (Ikeuchi *et al.*, 2013).

**Table 2:** Frequency of CRISPR/Cas9‐induced targeted mutations in hairy root clones, regenerated lines and progeny

	Transformation		Root clones					**Regenerated lines**					**Progeny**			
	Total # explants	# explants producing hairy roots	Total # screened	% with mutations	% chimeric	# used for regen	% capable of regen	Total # screened	% with stable mutations	% new mutations	% chimeric	Mutations (bp)	Total # screened	% mono‐allelic	% bi‐allelic	T‐DNA‐free
gPDSa + b U6.7SL	21	18	36	**92%**	**27%**	9	**33%**	12	**25%**	**67%**	**8%**	a2 (−11) a2 (−17) a1 (−25)	46	**93%**	**7%**	**4%**
gPDSa + b Csy4	32	28	27	**93%**	**20%**	5	**80%**	15	**27%**	**53%**	**20%**	a2 (−1) a1 (−3) a1, a2 (‐6) a1 (−8)				
gPDSa + b Csy4 + TREX2	38	35	47	**98%**	**30%**	14	**14%**	8	**0%**	**75%**	**25%**	a2 (−25[+7], −13 [+3]) a1 (−6)				
gPDSa + b Csy4 + D10A	35	30	24	**58%**	**14%**	2	**100%**	7	**100%**	**0%**	**0%**	a1 (−595) a1 (−364)				

**Figure 2:**
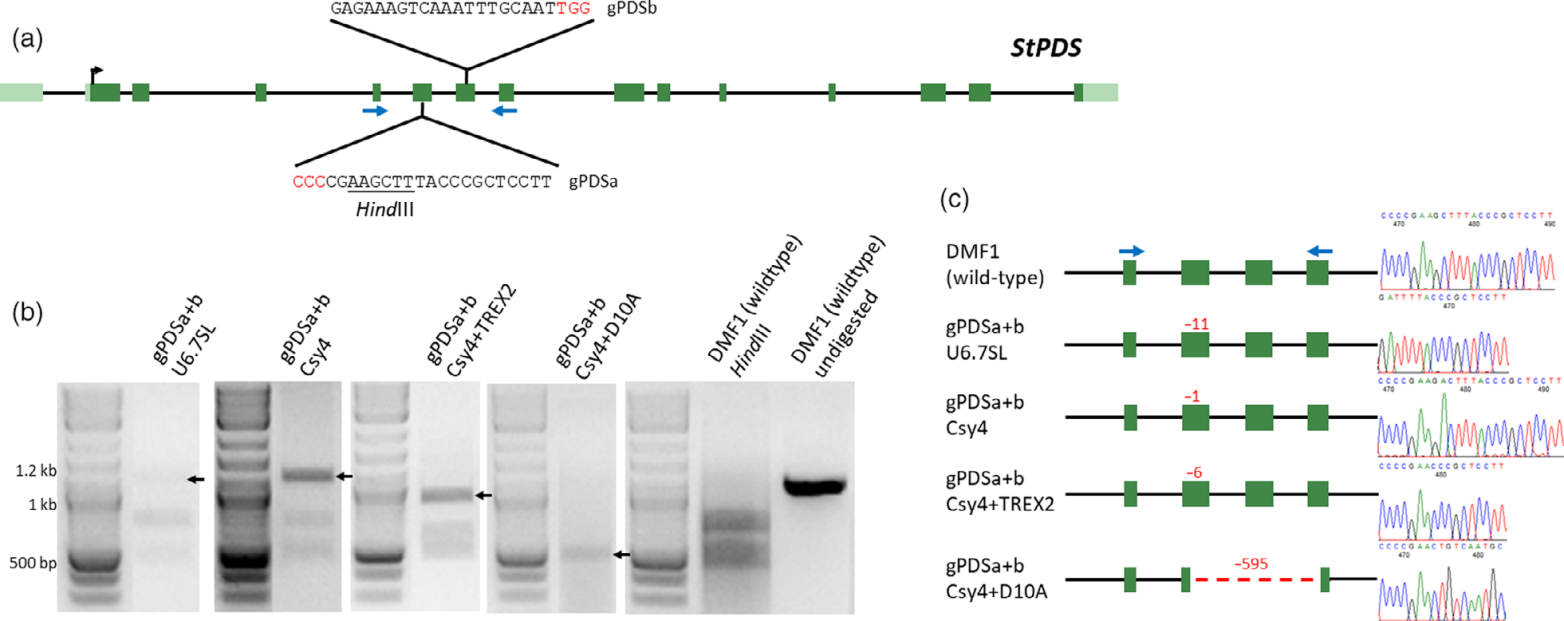
Targeted mutations in hairy root clones of potato using CRISPR/Cas9. (a) Schematic of potato *PHYTOENE DESATURASE* (*St*PDS) gene with guide RNA (gPDS) target sites. *Hind*III restriction enzyme site exists within gPDSa target site (underlined) used for detecting targeted mutations. Blue arrows indicate primers used for PCR‐based targeted mutation detection assays (see Methods). PAM sequences are in red. (b) PCR‐based targeted mutation detection assays for individual hairy root clones expressing CRISPR/Cas9 reagents. Paired‐guide RNAs (gRNAs) were used by co‐expressing gPDSa with gPDSb (gPDSa + b) within a single construct. gRNAs were expressed using individual U6 and 7SL RNA polymerase III (Pol III) promoters (U6.7SL), or a CaMV35S (35S) promoter using the CRISPR‐associated RNA endoribonuclease Csy4 (Csy4) system (Tsai *et al.*, 2014). Cas9, Cas9 nickase (D10A) and Csy4 were expressed using a 35S promoter, while the Trex2 exonuclease (TREX2) was driven by the FMV promoter (Cermák *et al.*, 2017). A 1037‐bp amplicon is expected with 417‐bp and 620‐bp products from *Hind*III digestion of wild‐type DNA to detect mutations within the gPDSa target site. gPDSa + b Csy4 + D10A amplicons were not digested. Arrows indicate bands containing targeted mutations used for cloning mutant alleles. (c) Sequencing of mutant alleles from individual hairy root clones expressing CRISPR/Cas9 reagents. Deletions (−) are shown with values in red. Blue arrows indicate primers used for sequencing. Black arrows indicate paired gRNAs. Right shows sequencing chromatographs of mutations. Hairy root clone numbers 2, 2, 8 and 10 are shown for gPDSa + b U6.7SL, Csy4, Csy4 + TREX2 and Csy4 + D10A, respectively (Table S3).

Stem explants were infected with *A. rhizogenes* cultures carrying CRISPR/Cas9 reagents (Total # explants), and explants forming hairy root clones were counted (# explants producing hairy roots) (Figure 1A). Hairy root clones carrying CRISPR/Cas9 reagents (total # screened) were screened for mutant alleles using a PCR‐based targeted mutation detection assay (% with mutations) and mutational chimerism (% chimeric) within the gPDSa target site (Figure 2). Data come from mutations in the gPDSa target site since no mutations were detected exclusively within the gPDSb target site. Hairy root clones with non‐chimeric mutations were used for regeneration (# used for regen), and hairy clones capable of regeneration (% capable of regen) were used to create regenerated lines. Regenerated lines derived from individual hairy root clones (# of root clones) were screened for mutant alleles from progenitor hairy root clones (% with stable mutations) (Figure S3). Mutant alleles cloned from hairy root clones and regenerated lines are given as deletions (−) or insertions [+]. Mutant alleles were clones from both alleles derived from the DM parent (a1) and M6 parent (a2) and listed from regenerated lines (Mutations). Progeny derived from gPDSa + b U6.7SL root clone 2 and regenerated line 2 (Table S3) were screened (total # screened) for mono‐allelic (% mono‐allelic), bi‐allelic (% bi‐allelic) germ‐line mutations, and T‐DNA integration using left border‐specific primers 5′‐TGGCAGGATATATTGTGGTGT‐3′ and 5′‐TACATTAAAAACGTCCGCAATGT‐3′ and recommended PCR conditions. Chimerism was determined if more than two alleles were cloned from a single event (see Methods). Percentages are based on total # screened. New mutations in regenerated lines (% new mutations) were determined if the mutations cloned from a regenerated line differed from the hairy root clone it originated from (Table S3). Data come from four independent transformation experiments for each CRISPR/Cas9 reagent.

Bold percentages are of total values in each category.

Regenerated lines from individual GUS‐expressing hairy root clones were assayed for GUS expression (Figure 1d‐f). Indeed, most of the regenerated lines (12 out of 13) from a single hairy root clone were positive for GUS activity, demonstrating transgene function persisted through regeneration in the absence of hygromycin selection (Table S2). For this reason, selection was not used during regeneration in future experiments (see Methods). Regenerated lines were further evaluated in glasshouse experiments (Figure S1). Regenerated lines demonstrated extensive root growth and supported vigorous shoots with fertile flowers capable of setting seed similar to wild type (Figure S1A‐B). However, in some instances, a more severe ‘hairy root phenotype’ could be observed with short internodes, small, curled leaves and delayed flowering (Figure S1C). Regenerated lines with a severe hairy root phenotype accounted for two of the 44 regenerated lines (4%) analysed and were omitted from further study (Table S3). All regenerated lines from hairy root clones had lower tuber yields than wild type (Figure S1D), putatively due to the energy investment in root production and hormone imbalances (De Vries‐Uijtewaal *et al.*, 1988).

### First‐generation targeted mutagenesis using hairy root clones

The use of hairy root *A. rhizogenes* stains enabled transformation of the seemingly *Agrobacterium* recalcitrant self‐compatible, diploid DMF1 genotype of potato. The consistency of GUS expression in regenerated lines from a single hairy root clone suggested that it would be possible to use this system to develop CRISPR/Cas9‐mediated mutants. To test this possibility, guide RNAs (gRNAs) were designed to target the potato *PHYTOENE DESATURASE* gene (*StPDS*; PGSC0003DMG400007542; Figure 2a) and assembled into binary vectors using methods developed by Cermák *et al. *(2017) (Figure S2, Table S1). Paired gRNAs (gPDSa and gPDSb) were designed to target exon VI containing a *Hin*dIII restriction enzyme site and exon VII in a ‘head‐to‐head’” orientation, respectively, for PCR‐based targeted mutation detection (Figure 2a‐b).

The paired gRNAs were expressed using four different gRNA expression constructs: separate Arabidopsis RNA polymerase III (Pol III) promoters (‘U6/7SL’), polycistronic mRNAs processed post‐transcriptionally by the CRISPR‐associated RNA endoribonuclease Csy4 from *Pseudomonas aeruginosa* using the D10A Cas9 nickase (’Csy4 + D10A’), or the Cas9 nuclease without (“Csy4”), or with the Trex2 exonuclease (“Csy4 + TREX2”) (Figure S2). The Cas9 nuclease, nickase, and Csy4 gRNA cassettes were driven by a 35S promoter, while Trex2 was driven by the FMV promoter (Cermák *et al.*, 2017). Expression constructs were delivered to DMF1 explants using the MSU440 strain, and transgenic hairy root clones were screened for mutations using PCR amplification of the target site (Figure 2b; Csy4 + D10A) or a PCR digest assay for mutations within the gPDSa target site (Figure 2b; other treatments). Interestingly, no mutations were detected within the gPDSb target site of any events or progeny carrying targeted mutations within the gPDSa target site apart from deletions spanning both gRNA target sites created by the Csy4 + D10A construct. This suggests gPDSb was effective in functioning in combination with gPDSa to create double‐stranded breaks via the Cas9 nickase but was ineffective in creating double‐stranded breaks alone once a targeted mutation was present in the gPDSa target site (Table 2). Hence, hereafter, mutations within the gPDSa target site will be referred to.

**Figure 3:**
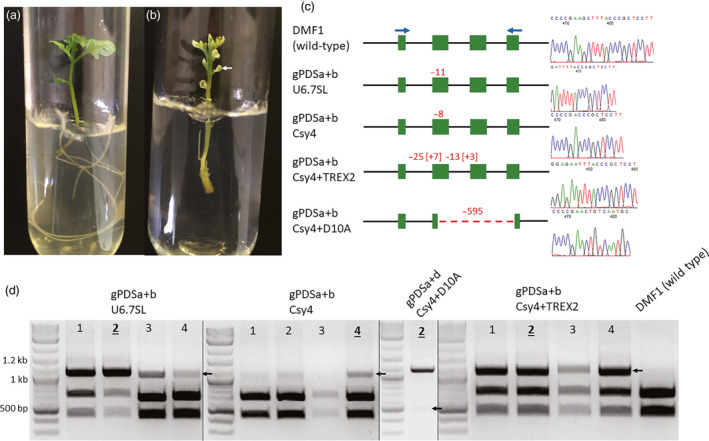
Targeted mutations in lines regenerated from CRISPR/Cas9‐expressing hairy root clones. (a) Wild‐type phenotype of DMF1 potato genotype grown in tissue culture. (b) Mono‐allelic photobleaching phenotype expressing in line regenerated from an individual hairy root clone carrying a mutant *St*PDS allele. Example from gPDSa + b U6.7SL root clone 2, regenerated line 2 (Table S3). Arrow indicates region of partial photobleaching. (c) Sequencing of mutant alleles from regenerated lines derived from hairy root clones. Deletions (−) and insertions [+] are shown in *St*PDS target region schematic with values in red. Blue arrows indicate primers used for PCR (d) and sequencing. Far right shows sequencing chromatographs of mutations. (d) PCR‐based targeted mutation detection assays for lines regenerated from CRISPR/Cas9 expressing hairy root clones. A 1037‐bp amplicon is expected with 417‐bp and 620‐bp products from *Hind*III digestion of wild‐type DNA. gPDSa + d Csy4 + D10A amplicons were not digested. Arrows indicate bands containing targeted mutations used for cloning mutant alleles. Regenerated lines originating from hairy root clones from Figure 2 are shown (Table S3). Mutations for lines underlined and bolded are shown in c.

Targeted mutations were detected and cloned in both transgenic hairy root clones (Figure 2c) and regenerated lines (Figure 3c) in all treatments (Table 2). Targeted mutations ranged from single base pair insertions (Csy4 + Trex2) to a 595‐bp deletion (Csy4 + D10A) (Table 2; Mutations), with generally high mutation rates across expression constructs, ranging from 64% (Csy4 + D10A) to 98% (Csy4 + TREX2) (Table 2; % with mutations). Although Csy4 + TREX2 yielded the highest percentage of hairy root clones with targeted mutations (98%), mutations were transmitted to regenerated lines as stable mutations at the lowest frequency (0%). Furthermore, the percentage of hairy root clones capable of regeneration was lowest with Csy4 + Trex2 than with other treatments (Table 2; % capable of regen). The higher targeted mutation rate, potential toxicity during regeneration and reduction in stable mutations using the Csy4 + Trex2 expression construct are presumably due to the higher mutagenetic activity provided by the Trex2 exonuclease in hairy root clones and opportunity for additional mutagenesis during the regeneration process. Nevertheless, using Trex2 could provide opportunities if a high efficiency of targeted mutagenesis is desired (Cermák *et al.*, 2017; Fauser *et al.*, 2014).

Stable mutations varied greatly across constructs (0%–100%; averaging 38%), with Csy4 + D10A having the highest (100%) and U6.7SL and Csy4 constructs averaging 26% (Table 2; % with stable mutations). A previous study reported chimerism in potato primary events using conventional *Agrobacterium* ranging from 50 to 100% and an even lower rate of stable mutations after the first clonal generation (40%) (Butler *et al.*, 2015). Applying a hairy root clone intermediate seemed to reduce chimerism across constructs in primary events (root clones, 14‐30%) and even more so in regenerated lines (0‐25%) (Table 2). The construct with the lowest chimerism in root clones, Csy4‐D10A (14%), also had the highest rate of stable mutations in regenerated lines (100%) (Table 2). The improved stability of mutations using the Csy4‐D10A construct could be explained by the large deletions generated using the D10A Cas9 nickase and the abolishment of the target PAM site (Figure 3c). Combining hairy root transformation with the Cas9 nickase could provide opportunities for improving first‐generation stable transmission and isolation of targeted mutations (Cermák *et al.*, 2017; Chiang *et al.*, 2016).

### Inheritance of germ‐line mutations and photobleaching phenotype in regenerated lines

Hairy root clones carrying non‐chimeric targeted mutations were regenerated into lines and analysed for targeted mutations (Figure 3, Table S3). Regenerated lines that shared mutations with the original hairy root clone were considered to carry stable mutations (Figure 3c; gPDSa + b U6.7SL and Csy4 + D10A), while others carried new mutations (Figure 3c; gPDSa + b Csy4 and Csy4 + TREX2). In either case, all analysed regenerated lines carried targeted mutations (Figure 3d). Regenerated lines with disruptive targeted mutations were capable of supporting a partial photobleaching phenotype in a mono‐allelic state (Figure 3b; arrow, Table S3; 62% of total lines), whereas regenerated lines with non‐disruptive or chimeric targeted mutations resembled wild‐type phenotypes (Figure 3a, Table S3; 38% of total lines).

**Figure 4:**
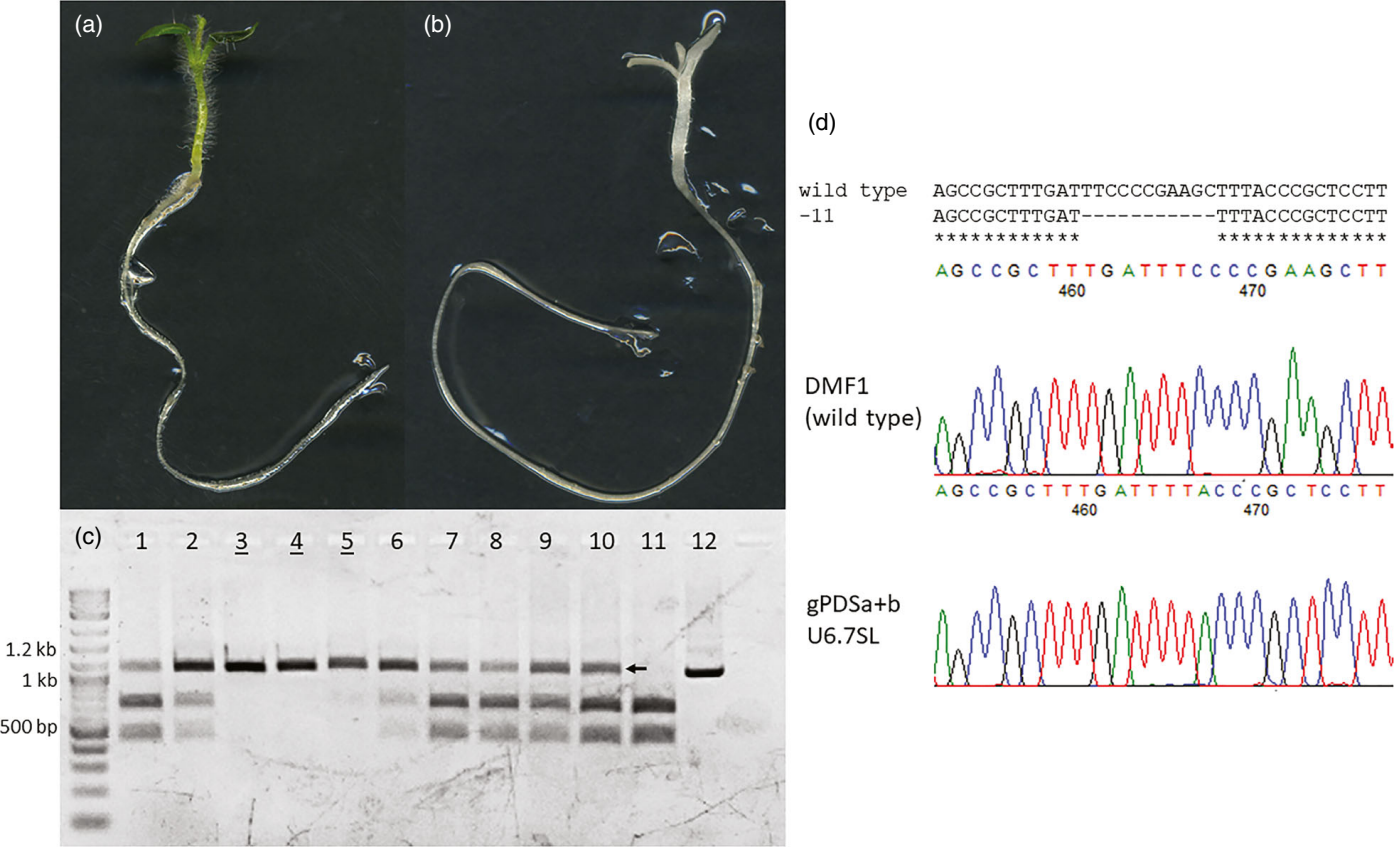
Targeted mutations in progeny derived from a single regenerated event expressing CRISPR/Cas9. (a) Wild‐type phenotype of DMF1 potato F2 seedling. 2 weeks. (b) Bi‐allelic photobleaching phenotype in progeny seedling derived from a CRISPR/Cas9 expressing regenerated event 2 weeks. (c) PCR digest assays for progeny derived from a single regenerated event expressing CRISPR/Cas9. Lanes 1‐2, and 6‐10 represent mono‐allelic mutants, while lanes 3–5 represent bi‐allelic mutants (underlined). Lanes 11 and 12 represent digested and undigested wild‐type, respectively. Arrows indicate amplicons resistant to *Hind*III digestion that were used for cloning the mutant allele. (d) Sequencing of mutant alleles from progeny derived from regenerated lines. Top shows alignment of 11‐bp deletion mutant allele detected in progeny. Bottom shows sequencing chromatograph of mutation. Example from gPDSa + b U6.7SL root clone 2, regenerated line 2 (Table S3).

Regenerated lines with stable mutations should be capable of germ‐line transmission to progeny and the creation of mono‐ and bi‐allelic mutations. To test this, a regenerated line created using the U6.7SL construct carrying a 11‐bp deletion was planted in a glasshouse and self‐pollinated to generate progeny (Figure 4, Table S3; bolded line). Progeny seedlings were monitored for photobleaching and sampled for PCR digest assays to detect targeted mutations and T‐DNA integration. As expected, both photobleached and wild‐type like progeny seedlings were observed (Figure 4a,b). When photobleached seedlings were sampled, no wild‐type alleles could be detected and the 11‐bp deletion allele was found in a bi‐allelic state (Figure 4c; underlined). In contrast, wild‐type like seedlings contained both wild‐type and 11‐bp deletion alleles in a mono‐allelic state (Figure 4c; 1‐2, 6‐10). Interesting, no wild‐type progeny were discovered in the population, putatively due to the persistent action of Cas9 in the germ‐line, and T‐DNAs segregated out of 4% of all progeny. These results confirm the formation of germ‐line mutations in hairy root clones that are capable of vegetative transmission via regeneration and inheritance to progeny through self‐pollination.

Genome editing has the potential to transform the way breeders approach crop improvement but is limited by the genotypes capable of being transformed and the efficiency in which mutants can be generated (Yin *et al.*, 2017; Zhang *et al.*, 2018). This is especially true in potato, in which vital, diploid, self‐compatible germplasm has been found to be recalcitrant to conventional *Agrobacterium*‐mediated transformation but is posed to be a valuable resource for breeding and functional genomics (Jansky *et al.*, 2014; Leisner *et al.*, 2018). We demonstrate the utility of *A. rhizogenes* strains for rapidly generating stable mutations within hairy root clones in potato genotypes recalcitrant to *A. tumefaciens* and regenerating fertile lines capable of fixing targeted mutations and segregating out T‐DNA insertions. The ability of hairy root clones to grow indefinitely in tissue culture readily provides transgenic material for regeneration and production of additional mutants when needed (Alok *et al.*, 2018; Ron *et al.*, 2014). This novel approach shines a new light on applications for hairy root transformation and simultaneously addresses major limitations for uses of genome editing for crop improvement.

## Methods

### Plant materials

The diploid, self‐compatible F1 hybrid DM1‐3xM6 (‘DMF1’) was used in the study (Endelman and Jansky, 2016). DMF1 was produced from a cross between the doubled‐monoploid DM1‐3 516 R44 (‘DM’) *S. tuberosum* Group Phureja line used to construct the potato reference genome (Potato Genome Sequencing Consortium, 2011) and M6, an inbred *S. chacoense* line with high fertility. M6 is homozygous for a dominant self‐incompatibility inhibitor, and its genome has been sequenced (Jansky *et al.*, 2014; Leisner *et al.*, 2018) DMF1 produces purple‐skinned tubers and vigorous vines (Figure S1A‐B). Three‐ to four‐week‐old tissue culture plants used for *Agrobacterium*‐mediated transformation were grown in 25 mm × 150 mm, round‐bottom, culture tubes on light racks set to 24‐h light photoperiod at 22C. Glasshouse grown plants were transplanted from tissue culture and grown for eight to ten weeks to be used for selfing under the same photoperiod and temperature as tissue culture plants. Fruit was harvested three weeks following fruit set.

### CRISPR/Cas9 reagent preparation

CRISPR/Cas9 cloning was conducted using vectors and Golden Gate assembly methods developed by Cermák *et al.*, 2017. For vector details, see Table S1.

### *Agrobacterium*‐mediated transformation

*Agrobacterium tumefaciens*‐mediated (*A. tumefaciens*) transformation using stem explants was conducted as previously described (Cearley and Bolyard, 1997). *Agrobacterium rhizogenese*‐mediated (‘hairy root’) transformation and regeneration were conducted as described by Cearley and Bolyard (1997) with the following modifications. Approximately 20‐40 stem explants approximately 1 cm in length were used per transformation experiment. Freshly prepared explants were inoculated in MS20 liquid media (Phytotechnology, Shawness Mission, KS; product number M524, 20% sucrose, pH 5.7) containing 1.5 mL overnight *Agrobacterium rhizogenese* culture (centrifuged at 3380 *g* and resuspended in MS20 liquid media). Inoculated explants were placed on MS20 solid media for a 48‐h co‐incubation period in the dark at 22C. After co‐incubation, explants were transferred to MS20 media containing plant selection (2, 5, 8 and 10 mg/L hygromycin) for generation of hairy root clones under the same conditions that tissue culture plants were grown (see Plant Materials) but under four layers of cheesecloth. Hairy root clones capable of growth on 8 mg/L hygromycin were used for PCR‐based targeted mutation detection, regeneration and/or GUS staining assays.

### Regeneration of hairy root clones

Regeneration of selected hairy root clones was carried out by transferring root tips (approximately 1–2 cm) to MS20 solid media, allowing growth for 1 to 2 weeks in the dark at 22C and preparing approximately 1‐cm hairy root explants for regeneration on MS20 media supplemented with zeatin riboside (9.56 mg/L, Phytotechnology; product number Z899), IAA (0.01 mg/L, Phytotechnology; product number I885) and GA_3_ (0.1 mg/L, Phytotechnology; product number G500) for four to six weeks under cheesecloth and growth conditions used for tissue culture plants (see Plant Materials). Approximately 50–60 root clone explants were used per regeneration experiment, and regenerated lines were rooted and grown under conditions used for tissue culture plants (see Plant Materials). Non‐chimeric hairy root clones were used for regeneration with no plant selection. Two to four regenerated lines were evaluated per hairy root clone.

### GUS staining and mutation characterization

GUS staining assays of hairy root clones and regenerated lines were conducted using previous methods (Butler and Hannapel, 2012) and visualized using a CanoScan LiDE 110 flatbed scanner (Cannon, Melville, NY). PCR amplicons were generated using primers 5′‐GTAGCTGCATGGAAAGATG‐3′ and 5′‐CTGAAGAAACCTGTTCAATG‐3′ with the Phire Hot Start II DNA polymerase (Thermo Fisher Scientific, Waltham, MA) and digested using the *Hind*III restriction enzyme (New England Biolabs, Ipswich, MA) under recommended conditions to detect mutations within the gPDSa target site (Figure 2a). Resistant bands were purified from 2% agarose gels using QIAquick Gel Extraction Kit (Qiagen, Valencia, CA) and subcloned using the Topo TA Cloning Kit (Life Technologies, Grand Island, NY) for Sanger sequencing at the University of Wisconsin Biotechnology Center. At least six colonies were sequenced per band for genotyping, and if more than two alleles were identified, the mutation was considered chimeric.

## Conflict of Interest

The authors have no conflicts of interest.

## Author Contributions

NMB conceived and designed the experiments. NMB performed the experiments. NMB and JJ analysed the data. SHJ contributed to materials/breeding tools. NMB, SHJ and JJ wrote the paper.

## Supporting information


**Figure S1** Phenotype of DMF1 wild‐type and lines regenerated from hairy root clones.
**Figure S2** Schematic of T‐DNAs used for delivering CRISPR/Cas9 reagents.
**Table S1** Vectors used for Golden Gate assembly of CRISPR/Cas9 reagents.
**Table S2** Frequency of GUS‐expressing hairy root clones and regenerated lines derived from *A. rhizogenes* infected stem explants.
**Table S3** Targeted mutation alleles cloned from hairy root clones and regenerated lines expressing CRISPR/Cas9.

## References

[pbi13376-bib-0001] Alok, A. , Kumar, J. and Upadhyay, S.K. (2018) Engineering in hairy roots using CRISPR/Cas9‐mediated editing In Hairy Roots (Srivastava V., Mehrotra S. and Mishra S., eds.), Singapore: Springer.

[pbi13376-bib-0002] Birhman, R.K. and Hosaka, K. (2000) Production of inbred progenies of diploid potatoes using an S‐locus inhibitor (*Sli*) gene, and their characterization. Genome, 43, 495–502. 10902714 10.1139/g00-012

[pbi13376-bib-0003] Butler, N.M. and Douches, D.S. (2016) Sequence‐specific nucleases for genetic improvement of potato. Am. J. Potato Res. 93, 303–320.

[pbi13376-bib-0004] Butler, N.M. and Hannapel, D.J. (2012) Promoter activity of polypyrimidine tract‐binding protein genes of potato responds to environmental cues. Planta, 236, 1747–1755. 22868575 10.1007/s00425-012-1726-7

[pbi13376-bib-0005] Butler, N.M. , Atkins, P.A. , Voytas, D.F. and Douches, D.S. (2015) Generation and inheritance of targeted mutations in potato (*Solanum* *tuberosum* L.) using the CRISPR/Cas system. PLoS ONE, 10, e0144591. 26657719 10.1371/journal.pone.0144591 PMC4684367

[pbi13376-bib-0006] Cearley, J.A. and Bolyard, M.G. (1997) Regeneration of *Solanum* *tuberosum* cv. Katahdin from Leaf Explants *in vitro* . Am. J. Potato Res. 74, 125–129.

[pbi13376-bib-0007] Cermák, T. , Curtin, S.J. , Gil‐Humanes, J. , Čegan, R. , Kono, T.J.Y. , Konečná, E. , Belanto, J.J. *et al* (2017) A multi‐purpose toolkit to enable advanced genome engineering in plants. Plant Cell, 29, 1196–1217. 28522548 10.1105/tpc.16.00922 PMC5502448

[pbi13376-bib-0008] Chiang, T.‐W.W. , le Sage, C. , Larrieu, D. , Demir, M. and Jackson, S.P. (2016) CRISPR‐Cas9D10A nickase‐based genotypic and phenotypic screening to enhance genome editing. Sci. Rep. 6, 24356. 27079678 10.1038/srep24356 PMC4832145

[pbi13376-bib-0009] Dangol, S.D. , Barakate, A. , Stephens, J. , Çalıskan, M.E. and Bakhsh, A. (2019) Genome editing of potato using CRISPR technologies : current development and future prospective. Plant Cell, Tissue Organ Cult. 139, 403–416.

[pbi13376-bib-0010] De Vries‐Uijtewaal, E. , Gilissen, L.J.W. , Flipse, E. , Sree Ramulu, K.S. and De Groot, B. (1988) Characterization of root clones obtained after transformation of monohaploid and diploid potato genotpes with hairy root inducing strains of *Agrobacterium* . Plant Sci. 58, 193–202.

[pbi13376-bib-0011] Endelman, J.B. and Jansky, S.H. (2016) Genetic mapping with an inbred line‐derived F2 population in potato. Theor. Appl. Genet. 125, 935–943. 10.1007/s00122-016-2673-7 26849236

[pbi13376-bib-0012] Fauser, F. , Schiml, S. and Puchta, H. (2014) Both CRISPR/Cas‐based nucleases and nickases can be used efficiently for genome engineering in *Arabidopsis thaliana* . Plant J. 79, 348–359. 24836556 10.1111/tpj.12554

[pbi13376-bib-0013] Halterman, D. , Guenthner, J. , Collinge, S. , Butler, N. and Douches, D. (2015) Biotech potatoes in the 21st century: 20 years since the first biotech potato. Am. J. Potato Res. 93, 1–20.

[pbi13376-bib-0014] Hirsch, C.N. , Hirsch, C.D. , Felcher, K. , Coombs, J. , Zarka, D. , Van Deynze, A. , De Jong, W. *et al* (2013) Retrospective view of North American potato (*Solanum* *tuberosum* L.) breeding in the 20th and 21st centuries. G3 3, 1003–1013. 23589519 10.1534/g3.113.005595 PMC3689798

[pbi13376-bib-0015] Ikeuchi, M. , Sugimoto, K. and Iwase, A. (2013) Plant callus: mechanisms of induction and repression. Plant Cell, 25, 3159–73. 24076977 10.1105/tpc.113.116053 PMC3809525

[pbi13376-bib-0016] Jansky, S.H. , Chung, Y.S. and Kittipadukal, P. (2014) M6: A diploid potato inbred line for use in breeding and genetics research. J. Plant Regist. 8, 195–199.

[pbi13376-bib-0017] Jansky, S.H. , Charkowski, A.O. , Douches, D.S. , Gusmini, G. , Richael, C. , Bethke, P.C. , Spooner, D.M. *et al* (2016) Reinventing potato as a diploid inbred line‐based crop. Crop Sci. 11, 1–11.

[pbi13376-bib-0018] Jia, H. and Wang, N. (2014) Targeted genome editing of sweet orange using Cas9/sgRNA. PLoS ONE, 9, e93806. 24710347 10.1371/journal.pone.0093806 PMC3977896

[pbi13376-bib-0019] Leisner, C.P. , Hamilton, J.P. , Crisovan, E. , Manrique‐Carpintero, N.C. , Marand, A.P. , Newton, L. , Pham, G.M. *et al* (2018) Genome sequence of M6, a diploid inbred clone of the high glycoalkaloid‐producing tuber‐bearing potato species *Solanum* *chacoense* *,* reveals residual heterozygosity. Plant J. 94, 562–570. 29405524 10.1111/tpj.13857

[pbi13376-bib-0020] Lowe, K. , Wu, E. , Wang, N. , Hoerster, G. , Hastings, C. , Cho, M.‐J. , Scelonge, C. *et al* (2016) Morphogenic regulators baby boom and wuschel improve monocot transformation. Plant Cell, 28, 1998–2015. 27600536 10.1105/tpc.16.00124 PMC5059793

[pbi13376-bib-0021] Marand, A.P. , Jansky, S.H. , Gage, J.L. , Hamernik, A.J. , de Leon, N. and Jiang, J. (2019) Residual heterozygosity and epistatic interactions underlie the complex genetic architecture of yield in diploid potato. Genetics, 212, 317–332. 30885982 10.1534/genetics.119.302036 PMC6499519

[pbi13376-bib-0022] Potato Genome Sequencing Consortium . (2011) Genome sequence and analysis of the tuber crop potato. Nature, 475, 189–195. 21743474 10.1038/nature10158

[pbi13376-bib-0023] Ron, M. , Kajala, K. , Pauluzzi, G. , Wang, D. , Reynoso, M.A. , Zumstein, K. , Garcha, J. *et al* (2014) Hairy root transformation using *Agrobacterium * *rhizogenes* as a tool for exploring cell type‐specific gene expression and function using tomato as a model. Plant Physiol. 166, 455–469. 24868032 10.1104/pp.114.239392 PMC4213079

[pbi13376-bib-0024] Tsai, S.Q. , Wyvekens, N. , Khayter, C. , Foden, J.A. , Thapar, V. , Reyon, D. , Goodwin, M.J. *et al* (2014) Dimeric CRISPR RNA‐guided FokI nucleases for highly specific genome editing. Nat. Biotechnol. 32, 569–576. 24770325 10.1038/nbt.2908 PMC4090141

[pbi13376-bib-0025] Veena, V. and Taylor, C.G. (2007) *Agrobacterium * *rhizogenes*: recent developments and promising applications. Vitro Cell Dev Biol – Plant, 43, 383–403.

[pbi13376-bib-0026] Yin, K. , Gao, C. and Qiu, J. (2017) Progress and prospects in plant genome editing. Nat. Plants, 3, 17107. 28758991 10.1038/nplants.2017.107

[pbi13376-bib-0027] Zhang, Y. , Massel, K. , Godwin, I.D. and Gao, C. (2018) Applications and potential of genome editing in crop improvement. Genome Bio. 20, 13. 10.1186/s13059-019-1622-6 PMC6335730 30651124

