# Supplementary material for: First‐generation genome editing in potato using hairy root transformation
Source: Plant Biotechnol J. 2020 Apr 16;18(11):2201–9. doi: 10.1111/pbi.13376 (PMC7589382; doi:10.1111/pbi.13376)

Supporting Information

**Supporting Figure 1**


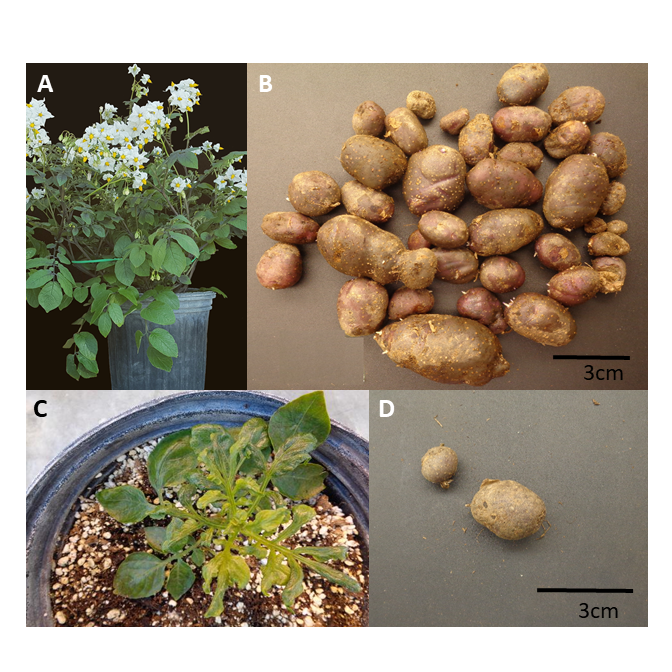


**Supporting Figure 2**


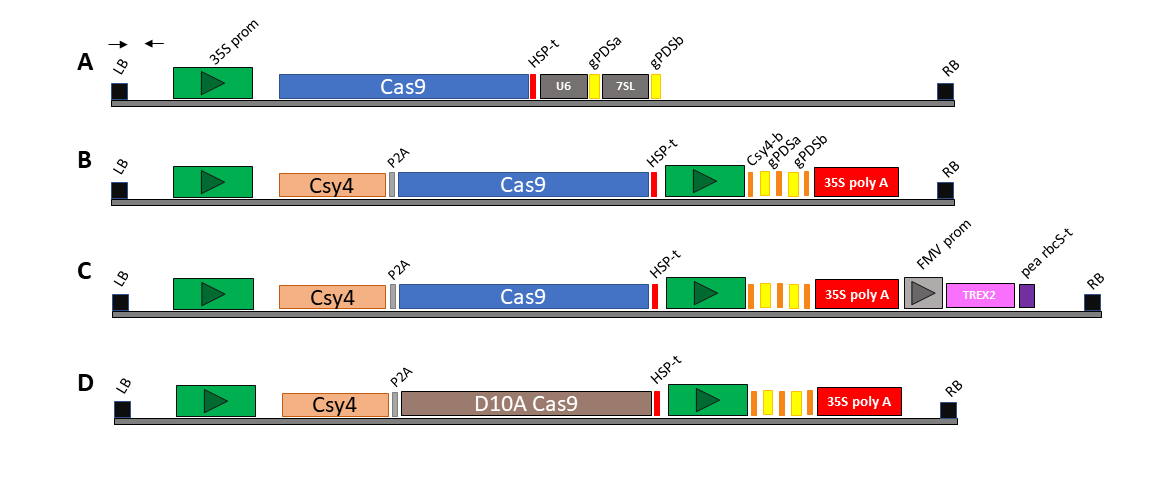


**Supporting Table 1**


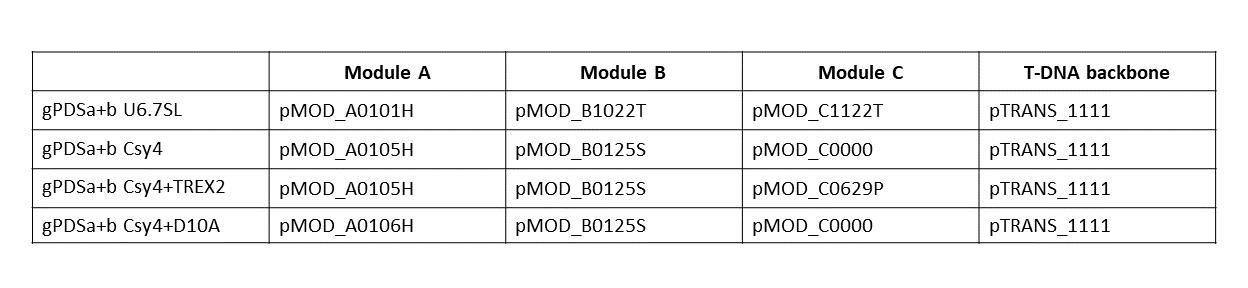


**Supporting Table 2**


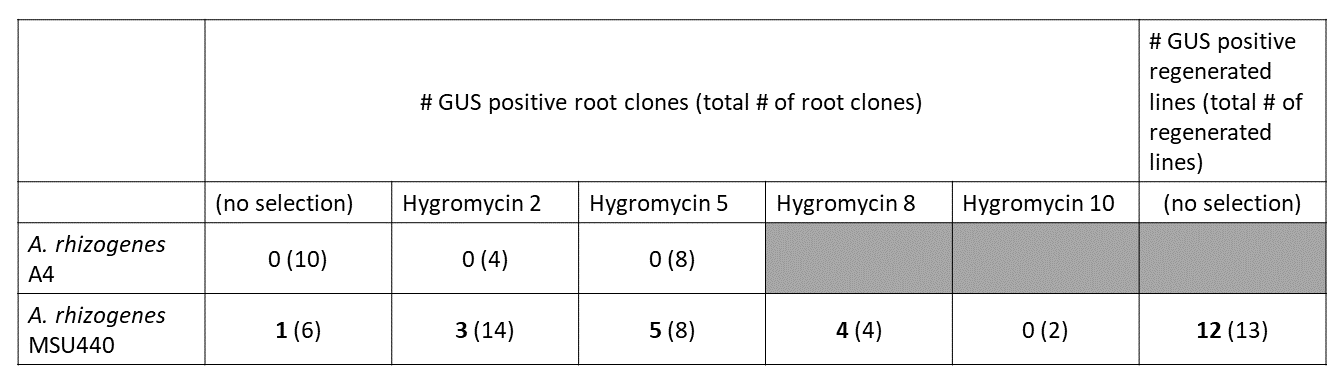


**Supporting Table 3**


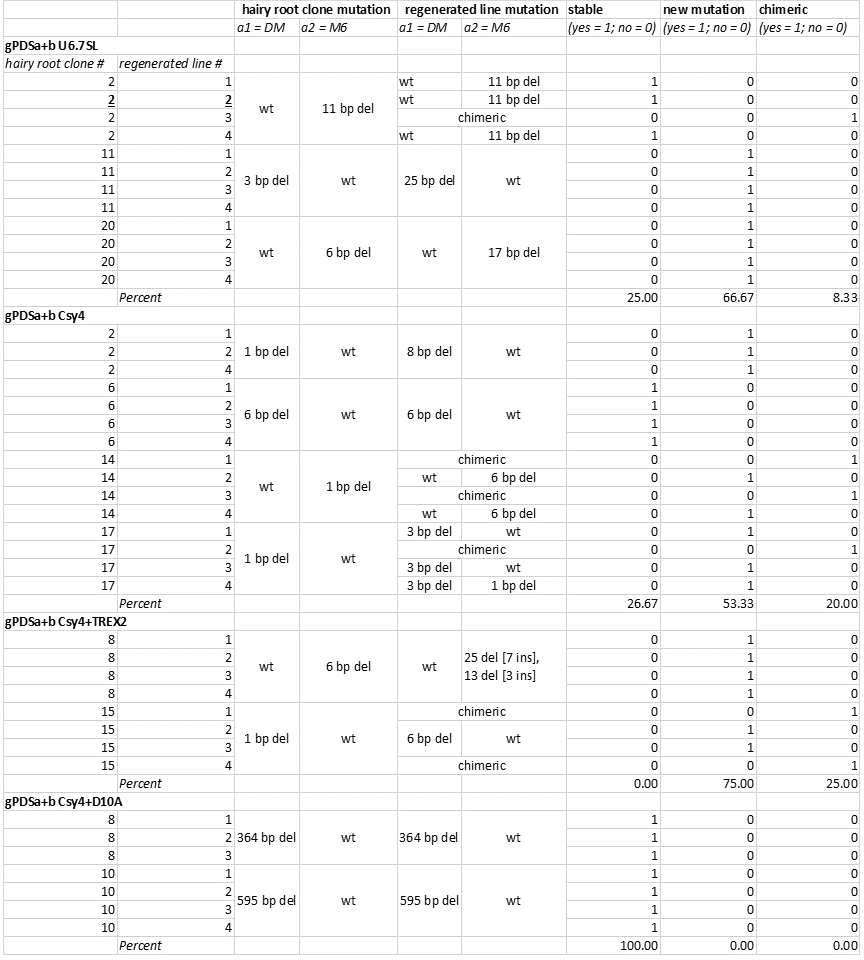

Supplement: Supplementary file 1 — Figure S1 Phenotype of DMF1 wild‐type and lines regenerated from hairy root clones. Figure S2 Schematic of T‐DNAs used for delivering CRISPR/Cas9 reagents. Table S1 Vectors used for Golden Gate assembly of CRISPR/Cas9 reagents. Table S2 Frequency of GUS‐expressing hairy root clones and regenerated lines derived from A. rhizogenes infected stem explants. Table S3 Targeted mutation alleles cloned from hairy root clones and regenerated lines expressing CRISPR/Cas9. [file PBI-18-2201-s001.docx]
